# Enuresis and Its Treatment

**Published:** 1896-07

**Authors:** 


### Selections and Abstracts.

### ENURESIS AND ITS TREATMENT.

One of the best articles on this subject that have recently appeared is that by Dr. M. Mendelsohn in the *Berliner Klinische Wochenschrift*, November 25 and December 9, 1895.

Enuresis means simply an involuntary discharge of the urine; hence every form of involuntary urination is enuresis. According to Foster's "Encyclopædic Medical Dictionary," we have:

Enuresis atonica—Enuresis from debility.
E. continua—Incontinence of urine both day and night.
E. diurna—Incontinence of urine by day.
E. irritata—Enuresis from irritability.
E. mechanica—Enuresis from mechanical causes.
E. nocturna—Nocturnal enuresis.
E. paralytica—E. associated with paralysis of bladder.
E. spastica—E. due to spasm of bladder.

Thus we have a term which may be applied to very different conditions. There is a wide difference between the condition represented in a child, with a bladder possessing a strong detrusor and a relatively weak sphincter vesicæ, who is unfortunate enough to suffer from enuresis nocturna, and that represented by the paralytic form whose bladder, incapable of being emptied voluntarily, the urine dribbles from overflow. And yet the term may be applied to either condition. In general, however, by enuresis we understand the condition found most frequently in children where there is nocturnal incontinence or, also, diurnal incontinence of urine, due essentially to a functional disturbance. The term incontinence is more apt to be applied to conditions in which there is a mechanical defect, and, according to Mendelsohn, we speak of "true incontinence" if the condition is due to defect of the sphincter, and of "false or paradoxical incontinence" where the palsied bladder-wall is at fault. In the former the bladder may be found to be always empty; in the latter, always full.

The purely functional condition where the urine and urinary apparatus are normal, which finds expression in nocturnal enuresis, is essentially a disease of childhood, and ordinarily ceases with the establishment of puberty. Out of thirty-two patients with this trouble Mendelsohn found only three were above the age of 14 years, and of these none had reached the age of 20. The greater number of cases were in males. The trouble may begin in the earliest years of life; that is, the child never learns to hold the urine, while ordinarily, by the time the teething process is over, children have learned to retain the urine for a sufficient interval. On the other hand, the trouble may begin in the later years of childhood, especially in children weakened by an attack of illness.

Mendelsohn thinks that too many theories have been constructed to explain this phenomenon; though there may be individual cases in which an underlying cause may be found, he believes that the phenomenon in the great majority of cases admits of a simple explanation. In many children the vesical sphincter is not sufficiently developed to be able, without the aid of the impulse of the will, to withstand the impulse communicated by the detrusor which has been stimulated to action, by the reflex, from the entrance of urine into the bladder-neck. This weakness passes off with the development of the prostate and the additional aid given by this organ in its intimate connection with the internal sphincter; and while in children the impulse to urinate comes at a certain distension of the bladder, by the entrance of urine into the bladder-neck —thus setting free a reflex stimulus of the detrusor muscle, the contraction of which and the consequent spontaneous emptying of the bladder may be successfully combatted during waking hours by the aid of the will and of the accessory cut-off muscle until a favorable opportunity—this sphincter in sleep may be unable alone to offer sufficient opposition. Thus children, who day after day are accustomed to urinate at relatively short intervals and at the first warning impulse, in the course of the disproportionately long sleep, are not apt to have that control which comes with practice during waking hours of holding the urine after an impulse coming from the reflex contraction of the detrusor has taken place. This combatting of the impulse of the contracting detrusor forms a closed circle; thus the bladder is distended to a certain point and urine enters the vesical neck. This gives the impulse to the detrusor, which on contracting throws more urine into the vesical neck, causing a reflex contraction of the sphincter, which overcomes the impulse from the detrusor.

There are two periods of the night at which the involuntary act is apt to take place—in the early portion of the night, during deep sleep, and the child does not wake at the accident; or at a later period, near dawn, when a longer time has elapsed. At this time, though sleep is less profound, the warning of a full bladder is apt to cause dreams, in which the child is aware of the desire to urinate, and, while dreaming that he has risen, or that the opportunity is present, relaxes the sphincter, and is then apt to awaken only to find a copious discharge of urine in the bed. Some children may further urinate involuntarily twice during the night—first, at the earlier period, during the profound sleep, and then later, toward dawn. The shame which an older child would naturally feel, and the scorn and derision in which he is held, the treatment to which he is often apt to be subjected, only result in making him timid and in aggravating the difficulty. The condition may go on further, so that even during the day enuresis may occur.

For treatment Mendelsohn advises three essential plans :

1. Regulation of the amount of fluid that the child is to take and the time for taking. In severe cases no fluid is to be allowed after noontime; the supper should be plain and nourishing. Sometimes idiosyncrasies may be found in the matter of the kind of fluids which aggravate the trouble, but fluids containing alcohol and carbonic acid are essentially bad and should be prohibited. The child should be instructed in the regular performance of micturition at stated and regular intervals, and all other habits, hours for meals, and time of going to stool, should in like manner be regulated. In milder cases this is sufficient.

2. A further and very important aid is to raise the foot of the bed so that it stands considerably higher than the head. In this way the urine as it flows from the ureters occupies the posterior portion of the bladder, and gravitates toward the fundus, and is thus longer in entering the vesical neck. For this plan he claims excellent results, the enuresis quickly ceasing in many cases. He advises a continuance of this position for 8 to 14 days after cessation of the condition, and a gradual lowering of the bed to the horizontal, taking 8 to 14 days in its performance. A return to this method may again be necessary.

3. For medicine he recommends the tincture of rhus aromatica, 15-drop doses at noon, evening, and bedtime, and believes its efficiency consists in allaying the sensitiveness of the bladder-neck.

As additional treatment the faradic current in very weak dosage, applied to the bladder-neck, beginning with a current that can scarcely be perceived, and at no time so strong as to cause discomfort, he looks upon as a valuable adjunct.

Mendelsohn mentions the treatment by belladonna in increasing dosage, as laid down by Trousseau, and for some cases finds chloral hydrate of use.

All mechanical methods which have been recommended—as a knotted towel in the middle of the patient's back to keep him on the side; pressure to perineum, to penis; closing the prepuce by collodion, etc.—he characterizes as cruel and as having a bad effect upon the child's mental condition. He also condemns nitrate of silver to the deep urethra, but believes small soft Nelaton catheters to be useful in some cases The child should be guarded against petty tyranny and teasing. The general health should be supervised; fresh air, change of air, exercise, cold bathing, etc., may be found to be necessary. —*American Medico-Surgical Bulletin.*

